# Shift Work and Breast Cancer

**DOI:** 10.3390/ijerph17249544

**Published:** 2020-12-20

**Authors:** Sarah Gehlert, Mark Clanton, on behalf of the Shift Work and Breast Cancer Strategic Advisory Group

**Affiliations:** 1 Suzanne Dworak-Peck School, University of Southern California, Los Angeles, CA 90089, USA; 2 Parkland Community Health Plan, Dallas, TX 75247, USA; mclanton@mac.com

**Keywords:** shift work, employment, breast cancer

## Abstract

The rates of shift work outside of daylight hours have increased in recent years, and nighttime shift work is now considered a potential carcinogenic occupational exposure. Light at night exposure, lower melatonin production, and the production of stress-related mediators disrupt normal sleep–wake cycles. Women who work lower-wage jobs and part-time workers whose shifts are determined entirely by their supervisors (rotating shifts) may be subject to stress related to efforts to align childcare and other needs with the unpredictable nature of rotating shift work. The causal link between breast cancer and the sleep cycle or circadian disruption are yet to be established; however, disruption of the circadian cycles by light at night exposure or chronic exposure to stress-related mediators have all been linked to the increased risk of breast cancer. We review the existing literature on shift work and breast cancer, identify knowledge gaps, and suggest future directions for research.

## 1. Introduction

Rates of shift work outside of daylight hours have increased in recent years, and nighttime shift work is now considered a potential carcinogenic occupational exposure as a result of decreased melatonin levels caused by light at night exposure [1,2]. In Europe, about one in five workers is employed in shift work involving nighttime hours [3]. Workers with lower levels of education are more likely to be employed in jobs involving unpredictable shifts involving some nighttime shift work than those with higher levels of education [4]. Although the percentage of female workers is unclear, women are more likely to work in the service sector than the industrial sector and non day work is more common in the service sector [5]. Women with fewer financial means are less able to control aspects of their work lives such as when they work, and thus have to accept less-favorable night shifts and shifts that rotate between day and night shifts [6].

The causal link between breast cancer and nighttime shift work or shift work rotating between day and night has been suggested, but has not yet been established [7]. In vitro experimental studies of melatonin have demonstrated anti-metastatic and anti-tumor cell invasive effects on breast cancer cell lines, and further mouse studies have yielded an association between light exposure during biological night, disruption of the wake–sleep cycle and increased breast cancer tumor growth. A few studies have attempted to understand the effect of shift rotations, light exposure and melatonin levels. One such study revealed that rotating shift work can reverse that normal pattern of melatonin production and decrease the overall 24 h levels of melatonin production.

A separate line of research from a study at the National Toxicology Board, that focused on carcinogens in flight attendants and nurses, suggested an increased risk from night work [8].

Our review of evidence for a link between shift work and breast cancer revels several gaps in the literature. Whereas both epidemiological and murine model studies suggest several routes by which work at night might predispose a woman to breast cancer, there is a lack of studies that follow changes in women who work primarily at night, including part- and full-time workers who have little or no control over the scheduling of their shifts, and the development of breast cancer. Likewise, the work linking carcinogens in nurses and flight attendants who work at night remains nascent.

While many authors have posited a link between shift work and breast cancer and a separate body of investigation has looked at the psychological, behavioral, and broad health effects of shift work on women, the two bodies of research have failed to overlap over three decades of inquiry. Another important consideration is that breast cancer mortality is much higher among underrepresented minority and lower socioeconomic status women globally; women who are less likely to control the timing of their shifts. In the United States, for example, black women are 40% more likely to die from breast cancer than white women, although they are no more likely to develop the disease [9]. Likewise, area poverty has been associated with both breast cancer incidence and mortality [10]. Taking employment and work/life balance into account has the potential to contribute to our understanding of the social determinants of breast cancer and breast cancer disparities. Thus, our aim of this paper is to stimulate research on the link between shift work and breast cancer, including how inflexible shift work contributes to breast cancer disparities. We do this by reviewing literature on shift work and breast cancer, the psychosocial effects of shift work, and the differential effects of socioeconomic status on the outcomes of shift work.

## 2. State of the Literature

Numerous epidemiological studies have suggested that night shift work may increase breast cancer risk, although few of these largely observational studies have clearly documented the necessary disruption of the circadian rhythm that is required by the International Agency for Cancer Research designation of shift work as a category 2a carcinogen. Lastly, it is also important to understand how social factors such as socioeconomic status and gender interact with shift work to contribute to breast cancer disparities.

In 2007, the International Agency for Cancer Research (IARC) Monograph working group listed shift work that involves circadian disruption as a class 2a or probable carcinogen. This designation put shift work in the same category as anabolic steroids, nitrogen mustard, vinyl fluoride, and 62 other agents, considering them to be class 2a carcinogens [11]. The 2019 IARC Monographs meeting changed the title to Shift Work at Night to better describe the exposure and subsequently re-affirmed the exposures classification as 2a or probable carcinogen [12]. The causal links between nighttime shiftwork and breast cancer have yet to be established; however, light at night related to circadian rhythm disruption provides a plausible biological mechanism that connects cancer risk with nighttime shift work. Normal nighttime melatonin levels not only regulate the sleep–wake cycle, but also affect tumor cell differentiation, proliferation, and apoptosis and may affect breast cancer invasion through the modulation of specific signaling pathways. In vitro studies of melatonin have demonstrated anti-human breast cancer tumor effects [13]. For example, melatonin inhibits breast cancer cell invasion through down-regulation of the p38 pathway and inhibition of MMP-2 and MMP-9 expression and activity [14].

Workers who are subject to shift rotations can present a more complex model for understanding the health impact of light at night on the sleep–wake cycle and melatonin production. In an attempt to understand the effect of shift rotations, light exposure, and melatonin levels, Dumont and Lanctôt measured the 24-h urinary 6-sulfatoxymelatonin (aMT6s) and ambulatory light exposure during night shift and day/evening shift periods of 13 full-time rotating shift workers [15]. The authors found that the total 24-h urinary excretion of aMT6s were the same in all shift work groups. However, there was a reversal of the normal sleep-wake levels of aMT6s, which implied that melatonin levels were higher during the day and lower at night, the opposite of what is normally observed in the sleep–wake cycle. The same study found that, with the exception of the reversal in the normal day–night pattern of melatonin production, although the total aMT6s levels in all groups were the same, higher levels of light exposure during night work likely decreased total melatonin production. This supports the idea that circadian disruption, of which decreased melatonin production is one adverse effect, might mediate night shift work and cancer risk [15].

The circadian timing mechanism in mammals is controlled by the suprachiasmatic nuclei. Filipski et al. created a mouse model to evaluate the effect of severe circadian dysfunction (ablation of the SCN) on tumor progression in mice with implants of Glasgow osteosarcoma and pancreatic adenocarcinoma. Both tumor types grew 2–3 times faster in mice with SCN lesions than in mice who underwent sham surgery. The survival of the mice with SCN lesions was significantly shorter than that of mice who underwent sham surgery. This led the authors to conclude that when the circadian rhythms of mice are disrupted, the growth of malignant tumors of two types is accelerated. This suggests that a host’s circadian clock may have a role in the endogenous control of tumor progression [16].

Stevens and Zhu estimated that 2–10% of all mammalian genes are clock-controlled, which points to extensive circadian gene regulation [17]. Just as disruption of the circadian rhythm affects nighttime melatonin levels, disruptions in the sleep–wake cycle, such as those caused by nighttime shift work, may also affect circadian clock-controlled genes [18]. A number of studies that focus on the effects on disease risk of circadian gene knockouts (KO) in mice and polymorphisms in humans suggests that absent or altered function of circadian genes might increase the risk of some human diseases [19,20]. Zhu et al. studied a circadian gene and cancer risk. They found that a structural variant in the circadian gene *PER3* was significantly associated with increased risk of breast cancer and posited it as a potential biomarker for breast cancer [21]. This circadian–cancer connection was later confirmed by other authors, who showed genetic associations between the circadian genes *NPAS2*, *CRY2* and *CLOCK* and breast cancer risk [22,23,24,25].

### 2.1. Epidemiology

An association between breast cancer and night work has been suggested by the authors of cohort studies, case-control studies, and meta-analyses [26]. Causation in epidemiological studies is judged by the extent to which studies that were done on specific subpopulations, in which the time and space of exposure vary with the disease outcome across the entire population [27,28]. Stevens summarized epidemiological studies that suggest an association between night shift work and breast cancer, namely night shift work, women who are blind, longer sleep time, and low bedroom light [28]. Other authors reported that (1) blind women have a lower risk of breast cancer; (2) long sleep duration is associated with reduced risk of breast cancer; and (3) bedroom light level is associated with breast cancer risk [26,29,30].

The Breast Cancer Now Generations Study, which followed 113,000 women over a forty-year period, reported their findings on light at night exposure and breast cancer. When participants entered the study, from 2003 onwards, their exposure to light at night and sleeping patterns were established through questionnaires, classifying light levels in their bedroom as low (too dark to see your hand, or you wear a mask), medium (light enough to see your hand in front of you, but not to see across the room) or high (light enough to see across the room, but not read, or light enough to read). The study concluded that exposure to light at night does not increase breast cancer risk [26].

However, no dose–response relationship emerged among workers exposed to fewer than 20 years of night work [31]. Lee et al., based on a review of 21 original articles and 5 meta-analyses on the relationship between night work and breast cancer and the compensation criteria of Denmark, concluded that breast cancer in patients with high exposure to night work is an occupational disease, thus patients should be eligible for workers’ compensation. In Korea, where general working hours are longer and night shifts for shift workers more frequent than in European countries, a number of factors emerge as potentially salient. These include total working hours, frequency of night work, work schedules that rotate and rest periods after night shifts and co-exposure to other occupational carcinogens that accrue over years of employment in non-day shift work. The authors suggest interventions to reduce breast cancer risk, such as restrictions on the frequency of night shifts and exposure periods to night work [31].

Wegrzyn et al., using data from the Nurses’ Health Study I and II to examine associations between rotating night shift work and breast cancer risk, found a total of 9541 incident invasive breast malignancies over 24 years of follow-up [32]. Women in the Nurse’s health Study I who had 30 years or more of shift work failed to exhibit a higher risk of breast cancer compared with those who never did shift work, with the caveat that follow-up occurred primarily after retirement from shift work. In the Nurses’ Health Study II, which enrolled younger participants, women with 20 years or more of shift work at baseline had significantly higher risk. The authors concluded that long-term rotating night shift work is associated with a higher risk of breast cancer, particularly among women who performed shift work during young adulthood [27,30].

In summary, although numerous epidemiologic studies, including cohort studies, case-control studies, and meta-analysis, have reported a positive association between light at night exposure and breast cancer, other studies report no effect. In addition to the need to create coherence between study subpopulations and standardizing study methodology, the single biggest challenge may be the need to more precisely measure the actual disruption of the circadian rhythm and reflect that disruption against health outcome(s) in large cohorts.

There remain significant observational epidemiologic data, biological (melatonin levels) and genetic (clock gene function and alteration) studies that support the theory that light at night disrupts the circadian rhythm in women and predisposes them to a higher risk of breast cancer.

### 2.2. Low Wage Shift Workers and Breast Cancer

Although the causal link between shift work at night and increased risk of breast cancer is yet to be established, social factors such as socioeconomic status can contribute to breast cancer disparities. Coughlin completed an extensive literature review of the social determinants of breast cancer and found convincing evidence for a number of contributors to risk and survival [10]. Census-tract-level-poverty, for example, has been associated with late-stage diagnosis, which delays treatment and increases mortality [33].

Low socioeconomic status has also been associated with shift work. The report on the economics of workplace flexibility by the Council of Economic Advisors of the Executive Office of the President of the United States concluded that less skilled workers have less flexibility in terms of the scheduling of when they work their shifts than do more highly skilled workers [34]. Less skilled workers, who tend to be those of lower socioeconomic status, have less control over the shifts that they work. Workers with family incomes below the U.S. federal poverty level are less likely than higher-earning workers to have control over the timing of their shifts and employees entering the workplace between 7 a.m. and 10 a.m. tend to be better educated and to earn more than those arriving at work at other hours [4].

In July 2013, the Urban Institute released a report titled, “Nonstandard Work Schedules and the Well-Being of Low-Income Families” that described occupations and industries with the highest share of workers with non-standard schedules [35]. Occupations with high percentages of non-standard schedules included security guards, waiters/waitresses, nurses and home health aides, janitors and laborers. Industries with highest percentages include accommodation and food, arts and entertainment and retail, transporting and warehousing, health and social assistance [35]. Hansen noted the following sectors with high rates of night work—hospitals, hotels, transportation, security, and industries that depend on 24-h production schedules [36].

Schedule unpredictability occurs when the hours or days of work are controlled solely by employers without input from workers. Schedules that vary from day to day or from week to week [6] and schedule unpredictability occur when the hours or days of work are controlled solely by employers without input from workers. Schedules that vary from day to day or from week to week require flexibility on the part of workers. In 2012, almost three in five wage and salary workers, 75.3 million in total, were paid by the hour [36]. These low-income and part-time workers often are ignored in studies of the effects of shift work. In a survey of problematic scheduling among a large representative sample of early career adults (26 to 32 years of age) working hourly jobs, 41% reported knowing “when they need to work” one week or less in advance of the work week in question [6]. The percentage was even higher for part-time workers (47%).

While men are only somewhat more likely than women to have flexible schedules [37], McMenamin found that white shift workers were more likely than back or hispanic shift workers to have control over when their shifts occurred [38]. It is likely then, that underrepresented minority women would have less control over their shifts than white women. This again suggests differences among female shift workers according to race/ethnicity and socioeconomic status, due in part to the nature of the shift work that women with lower levels of education and income must assume, which is more likely to be in the service sector. Liu et al. found that the risk of cancer increases with years in night shift work for both men and women [39].

Women who work inflexible shifts are more likely to also assume primary child care duties, which is another major source of physiological stress. Lambert, Fugiel, and Henly (2014) found that 45% of mothers with children below the age of 12 years reported that their employers decided their schedule without their input and concluded that women are disproportionately affected by the necessity to arrange childcare [40].

In a seminal work on shift work and health, Finn was among the first to suggest that unpredictable shift work that is out of the control of workers interrupts endocrine and other physiological processes, and that rhythmic adjustments to new work schedules take days to weeks to occur. Consequently, the body is in a constant state of adjustment, with both physical and emotional consequences [41]. This almost certainly is compounded by work–life conflict [42], defined as stress associated with managing work and family responsibilities, such as finding last-minute childcare for working single mothers. In addition, unpredictable work schedules, such as last-minute changes of work shifts, may lead to income instability and such economic volatility may cause additional stress and conflicts for low-wage workers [43].

The physiological stress produced in low-wage workers by unpredictable work schedules and the work-life conflict that it produces (e.g., finding last minute childcare, interfering with establishing and maintaining partner or other social relationships, and arranging food shopping and transportation) suggests another route to breast cancer beyond that which comes from light exposure during night shifts. Linnenbringer, Gehlert, and Geronimus and others posit a physiological route through repeated demands on stress hormone system and the ultimate physiological “weathering” that it brings [1,44]. These authors suggested an effect on breast cancer subtype produced by prolonged exposures to social stressors like juggling childrearing and work demands. These social stressors that disproportionately affect women may add a layer of physiological disruption to that produced by disruption in melatonin production from light at night.

## 3. Future Directions for Research

The first direction for research suggested by this review is determining whether breast cancer risk is higher among low-wage hourly employees with histories of unpredictable shifts (i.e., those controlled by employers). This work will likely require the use of existing large datasets and secondary data analysis. Breast cancer data might come from state cancer registries. Employment data might come from the O*NET database, sponsored by the US Department of Labor and the Employment and Training Administration, contains detailed information on almost 1000 occupations. Workers’ responses to survey questions are used to create measures capturing a multitude of different aspects of an occupation, e.g., use of technology, physical demands, customer interaction, skills required—and many, many more. Researchers can add these measures to any dataset by matching on SOC (Standard Occupational Classification) codes, which are commonly used to classify jobs. This allows researchers to incorporate different aspects of job quality into the inquiry. Data bases in other countries can similarly be combined.

Should the link that is suggested by prior research be established, our review highlights the need for intervention(s) to address the known risk(s) associated with night shift work and test for intervention feasibility and acceptability. This likely will require original data collection and may require qualitative and quantitative approaches.

## 4. Conclusions

Although no direct association has been established between shift work and breast cancer risk, mounting evidence suggests that shift work contributes to breast cancer risk. This evidence comes from studies that use a variety of methods, from epidemiologic studies like the Nurse’s Health Study I and II, murine studies, and environmental studies converge to establish the need for further work in the area. Likewise, additional study is needed on how stress from challenges to lower wage workers’ work and life balance might further contribute to breast cancer risk is warranted.
